# To What Extent Can Orbital Cellulitis Emergency Aspect Influence the Diagnosis of Maxillary Sinus Lymphoma?

**DOI:** 10.1155/2020/2304861

**Published:** 2020-04-08

**Authors:** Yousra Ajhoun, Ismail Aissa, Taoufik Abdellaoui, Yasmine Chaoui Roqai, Ilias Benchafai, Redouan Messaoudi, Rachid Zerrouk, Karim Reda, Abdelbarre Oubaaz

**Affiliations:** ^1^Ophthalmology Department, Military Instruction Hospital Mohammed V, Rabat, Morocco; ^2^Anesthesiology and Intensive Care Department, Military Instruction Hospital Mohammed V, Rabat, Morocco; ^3^ENT Department, Military Instruction Hospital Mohammed V, Rabat, Morocco

## Abstract

We present a case of a 46-year-old woman admitted to the emergency department for acute swelling and erythema of the right eyelid for 3 days. Ophthalmological examination was notable for 10/10, P2 best visual acuity, and inflammatory periorbital edema, without exophthalmia nor extraocular motility disturbance. Intraocular pressure was 14 mmHg and fundoscopic examination was not notable for any abnormality. Preseptal cellulitis diagnosis was made, and oral antibiotherapy was immediately started; after 72 hours, the patient did not improve and started complaining of pain on ocular movements. Brain and orbit MRI scan revealed right retroseptal cellulitis associated with homolateral pansinusitis. Intravenous antibiotherapy with oral corticosteroid was started simultaneously leading to full remission but with steroid dependency; 5 days after finishing prednisone, orbital cellulitis symptoms reappeared. The same treatment protocol was given but with corticosteroid tapering over weeks. Nevertheless, steroid dependency persisted. Except for the inflammatory syndrome, complete biological examinations did not disclose any abnormalities. The patient underwent maxillary sinus and fat orbital biopsy; however, histopathological examination was not contributory. Persistence of steroid dependency, chronic atypical rhinosinusitis, normal paraclinical investigations, and age of patient let us suspect lymphoma origin hidden by chronic corticosteroid. We carried out for the second time a maxillary sinus biopsy after stopping steroids, which disclosed primitive non-Hodgkin lymphoma of the maxillary sinus. The aim of this observation is firstly to evoke though it is exceptional the diagnosis of maxillary lymphoma in case of atypical orbital cellulitis and secondly to incite clinicians to be more vigilant in prescribing corticosteroid even if there is an emergency character of orbital cellulitis.

## 1. Introduction

Orbital cellulitis is a real emergency with significant visual and central nervous system complications [1]. Its typical clinical presentation allows in most cases a fast and easy diagnosis [2]. However, the clinician should stay vigilant when considering infectious disease diagnosis. Indeed, although it is extremely rare, orbital cellulitis can hide a maxillary lymphoma and lead to late diagnosis [3].

In order to get an early resolution of the inflammation component, corticosteroids are often prescribed in addition to intravenous antibiotherapy in the treatment of orbital cellulitis. Nevertheless, what can be the impact of preliminary steroids in the diagnosis of underlying lymphoma?.

## 2. Case Report

**Figure 1:**
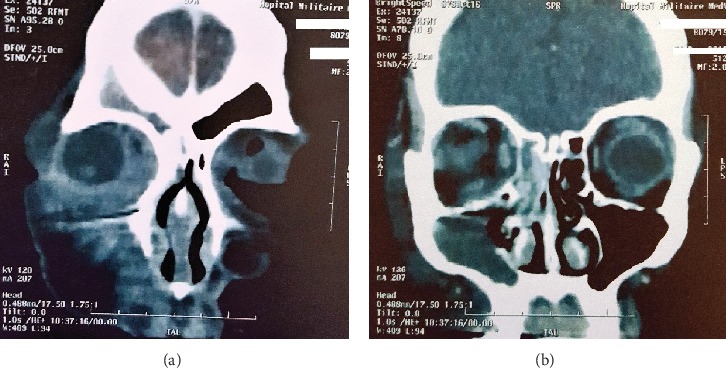
Orbitocerebral MRI scan in coronal sections showing extra and intraconical fat infiltration of the right orbit with filling of the right frontal sinus (a). Filling of the right maxillary and ethmoidal sinus (b).

A 46-year-old woman was admitted to the emergency department for acute swelling and erythema of the right eyelid for 3 days. Ophthalmological examination was notable for 10/10, P2 best visual acuity, and inflammatory periorbital edema grade 3, without exophthalmia nor extraocular motility disturbance. Normal intraocular pressure and normal fundoscopic examination. On the left eye, examination was unremarkable. There were no general signs. Preseptal cellulitis diagnosis was made and oral antibiotherapy was immediately started. But after 3 days, the patient did not improve and started complaining of pain on ocular movements. Brain and orbit MRI scan revealed a right retroseptal cellulitis associated with homolateral pansinusitis (Figure 1). Intravenous antibiotherapy with oral corticosteroids was started simultaneously in this way: vancomycin (40 mg/kg/day), ceftriaxone (100 mg/kg/day). The patient was shifted into oral antibiotics (ceftriaxone) on the 4th day and discharged on the 7th day. Prednisone was started with 1.5 mg/kg/day for 3 days followed by 1 mg/kg/day for another 3 days, then gradual tapering over 2 weeks.

The patient presented gradual improvement until full remission but developed steroid dependency. Five days after finishing prednisone, orbital cellulitis symptoms reappeared. An MRI scan performed for the second time found the same aspect without another abnormality. The same treatment protocol was given but with corticosteroid tapering over weeks. Nevertheless, steroid dependency persisted; thereby, a posology of 10 mg/day of prednisone had been remained. Except for the inflammatory syndrome, complete biological examination did not disclose any abnormality. Maxillary sinus biopsy found nonspecific chronic rhinosinusitis. Fat orbital biopsy revealed a slight inflammation of fat-connective tissue. Both biopsies did not find any vasculitis or tumoral signs.

The persistence of steroid dependency, chronic atypical rhinosinusitis, normal paraclinical investigations, and the age of the patient let us suspect an orbital inflammation secondary to rhinosinusal lymphoma, which is hidden by chronic corticosteroid therapy. Our attitude was to carry out for the second time maxillary sinus biopsy 15 days after stopping prednisone. As our expectation, the histopathological examination disclosed primitive non-Hodgkin lymphoma of the maxillary sinus, precisely diffuse large B cell lymphoma subtype. After normal extension assessment, the patient was transferred to the oncological department and R-CHOP regimen chemotherapy was started.

## 3. Discussion

Orbital cellulitis is defined as a serious infection of the orbital soft tissues that exist behind the orbital septum. It is commonly seen in children [4] and in almost 86% to 98% of cases correspond to a complication of bacterial rhinosinusitis especially in cases of pansinusitis [5]. This disease is a potentially life-threatening emergency [1]. All that puts pressure on urgent diagnosis and treatment. Yet not all orbital cellulitis symptoms result from an infectious origin. Indeed, though it is exceptional, maxillary lymphoma may mimic orbital cellulitis. To our knowledge, our observation describes the third case of orbital cellulitis revealing maxillary lymphoma [3, 6]. In fact, this tumoral entity is generally insidious with myriad presentations and it is revealed in most cases by sinonasal nonspecific signs [7].

Orbital cellulitis treatment consists of intravenous broad-spectrum antibiotics, corticosteroids, and treatment of associated sinusitis. To date, there are no clear recommendations concerning the use of corticosteroids in the acute management of orbital cellulitis [1]. Many authors support that glucocorticoids do not adversely affect the clinical outcome; in the opposite, it allows an early resolution of inflammation without altering the complete resolution of infection [1, 8]. Nevertheless, prescribing steroids in this urgent situation and guiding the therapeutic management depending on the response of steroidal anti-inflammatory drugs may be a real trap, since it can hide lymphomatous pathology. In fact, giving corticosteroids before biopsy and before diagnosing lymphoma may lead to clinical and morphological modifications and complexity on the process of reaching diagnosis [9]. Recently, Kan et al. have found, after studying the case of 31 patients who were given before biopsy corticosteroid agents, primary diagnosis difficulty in almost 50% of cases for DLBLC and 100% for low-grade B cell lymphoma, Hodgkin lymphoma, and T cell lymphoma [9]. Another study has found that preliminary corticosteroids may adversely affect the histopathological accuracy or lead to a late definitive diagnosis of mediastinal lymphoma [10]. Our case consolidates this fact; after two biopsies undergone without stopping corticosteroids, diagnosis could not be reached. After 15 days without steroids, maxillary sinus biopsy revealed primitive DLBCL.

## 4. Conclusion

It is rather imperative that physicians should keep in mind the diagnosis of maxillary lymphoma when encountering a case of orbital cellulitis, especially when it occurs in an adult person and when it does not respond to usual treatment.

Corticosteroids are a good help for resolving orbital cellulitis inflammation; however, if given in a hurry, they may hide the tumoral origin of inflammation and make the process of reaching the real diagnosis difficult.
